# Resource Allocation in the Cognitive Radio Network-Aided Internet of Things for the Cyber-Physical-Social System: An Efficient Jaya Algorithm †

**DOI:** 10.3390/s18113649

**Published:** 2018-10-27

**Authors:** Xiong Luo, Zhijie He, Zhigang Zhao, Long Wang, Weiping Wang, Huansheng Ning, Jenq-Haur Wang, Wenbing Zhao, Jun Zhang

**Affiliations:** 1 School of Computer and Communication Engineering, University of Science and Technology Beijing (USTB), Beijing 100083, China; G20178705@xs.ustb.edu.cn (Z.H.); B20180325@xs.ustb.edu.cn (Z.Z.); lwang@ustb.edu.cn (L.W.); weipingwangjt@ustb.edu.cn (W.W.); ninghuansheng@ustb.edu.cn (H.N.); 2 Beijing Key Laboratory of Knowledge Engineering for Materials Science, Beijing 100083, China; 3 Department of Computer Science and Information Engineering, National Taipei University of Technology, Taipei 10608, Taiwan; jhwang@csie.ntut.edu.tw; 4 Department of Electrical Engineering and Computer Science, Cleveland State University, Cleveland, OH 44115, USA; w.zhao1@csuohio.edu; 5 Science and Technology Division, North China Institute of Science and Technology, Beijing 101601, China; zhangjunnm@ncist.edu.cn

**Keywords:** cognitive radio networks (CRNs), orthogonal frequency division multiplexing (OFDM), resource allocation, Internet of Things (IoT), Jaya algorithm

## Abstract

Currently, there is a growing demand for the use of communication network bandwidth for the Internet of Things (IoT) within the cyber-physical-social system (CPSS), while needing progressively more powerful technologies for using scarce spectrum resources. Then, cognitive radio networks (CRNs) as one of those important solutions mentioned above, are used to achieve IoT effectively. Generally, dynamic resource allocation plays a crucial role in the design of CRN-aided IoT systems. Aiming at this issue, orthogonal frequency division multiplexing (OFDM) has been identified as one of the successful technologies, which works with a multi-carrier parallel radio transmission strategy. In this article, through the use of swarm intelligence paradigm, a solution approach is accordingly proposed by employing an efficient Jaya algorithm, called PA-Jaya, to deal with the power allocation problem in cognitive OFDM radio networks for IoT. Because of the algorithm-specific parameter-free feature in the proposed PA-Jaya algorithm, a satisfactory computational performance could be achieved in the handling of this problem. For this optimization problem with some constraints, the simulation results show that compared with some popular algorithms, the efficiency of spectrum utilization could be further improved by using PA-Jaya algorithm with faster convergence speed, while maximizing the total transmission rate.

## 1. Introduction

With the rapid development of the Internet of Things (IoT) within the cyber-physical-social system (CPSS), there is an ever-increasing demand for communication network bandwidth for achieving high-performance applications which enrich and broaden the interactions and connections in the cyber-physical-social world. Thus, some communication technologies have been developed using new frequency bands. Moreover, various improved modulation and coding technologies have also been proposed to utilize the existing frequency spectrum more efficiently [1,2,3]. However, spectrum resources are limited. They are a kind of extremely valuable natural resource and are becoming increasingly restricted or even depleted. In response to this limitation, it is necessary to improve the current spectrum management methods. Fortunately, the recent advances of big data and artificial intelligence technologies make it possible to address the issue.

Cognitive radio network (CRN), as an intelligent wireless communication system, can dynamically share and allocate the spectrum in accordance with the current state of the channel, and it has been seen as an effective solution to the current low utilization of spectrum resources [4,5]. Furthermore, CRNs have been employed to develop the high-performance IoT [6,7,8,9,10,11,12,13]. For example, CRN technology applied to wireless sensor network (WSN) systems in IoT can well sense the surrounding environment and adapt to environmental changes by changing the transmission parameters in real time through information acquired from the environment. Such characteristics make it especially suitable for practical production scenarios. Generally, in the CRN-aided IoT, the secondary user (i.e., cognitive user) may selectively access the idle frequency band of the primary user (i.e., authorized user) to improve the utilization of the licensed spectrum. Meanwhile, some limit requirements should be met by the interference of the secondary user to the primary user for the frequency band. These limits include the constraints of total power, the bit error rate (BER), and the acceptable interferences of the primary user [14].

Furthermore, orthogonal frequency division multiplexing (OFDM), as a multi-carrier parallel wireless transmission technology, has been identified as one suitable alternative to deal with the allocation and transmission of spectrum resources in CRNs [15,16]. Hence, the issue of how to adaptively allocate downlink resources in a cognitive multi-user OFDM system for IoT has drawn much attention from researchers.

Generally, there exist three types of CRNs, including interweave, underlay, and overlay CRNs. In an interweave scenario, the secondary users adaptively utilize the spectrum band whenever it is not used by the primary users [17]. In the underlay case, the secondary users are allowed to coexist with the primary users, provided that the interference caused to the primary users is below a predefined threshold [17]. In the latter category, the secondary system has the knowledge of the primary system codebook, channel gains, and transmitted information. A part of the secondary users’ power can be used to retransmit the primary users’ message so that the secondary users are able to maintain or improve the primary rate while achieving their own communication goals [18]. Generally speaking, the existing solutions mostly use the traditional mathematical optimization method or some greedy searching algorithms, which may suffer from a quite high computational complexity during the implementation process [18,19]. Some evolutionary algorithms, including simulated annealing (SA) [20], genetic algorithm (GA) [21], particle swarm optimization (PSO) [22,23], differential evolution (DE) [24], and immune clonal optimization (ICO) [25], are employed to deal with this issue, with the help of their effective computational features in the swarm intelligence paradigm. Through the use of those algorithms, a satisfactory solution effect was achieved during the resource allocation in CRNs [20,21,22,23,24,25]. However, there is still room to further improve the optimization effect due to the inherent disadvantages of those algorithms in the practical engineering applications of IoT, such as the possible ease of falling into the local optimal solutions in some cases, the difficulty adjusting many key parameters during the implementation process of the algorithms, and many drawbacks [26,27,28,29,30,31,32].

The Jaya algorithm has been widely used in engineering optimization fields due to its excellent searching ability with an algorithm-specific parameterless feature [33]. In consideration of the satisfactory optimization performance achieved by the Jaya algorithm, there is a general idea to utilize the Jaya algorithm to better serve CRN-aided IoT. In this article, motivated by our previous work in ref. [34], the power resource allocation in the downlink of a CRN-aided IoT system is modeled as a constrained optimization problem, and then a solution method is accordingly presented by incorporating the proposed Jaya algorithm into the cognitive OFDM radio network model to achieve a satisfactory power allocation result. Specifically, compared with the traditional Jaya, the proposed Jaya algorithm, named PA-Jaya, achieved faster convergence speed by using a parallel structure and an asynchronous iteration strategy. Actually, the simulation results show that compared with other popular optimization algorithms, our PA-Jaya algorithm can achieve a better power allocation with faster converge speed, which means that it can improve the efficiency of spectrum utilization more effectively in IoT.

This article is organized as follows. Section 2 presents the mathematical model used in the optimization problem we discuss. In Section 3, in addition to analysis for the basic idea and implementation of the traditional Jaya algorithm, the solution method using the proposed PA-Jaya is also presented. Section 4 shows the simulation results to verify the advantages of using the PA-Jaya algorithm, with a comparison with other algorithms. Finally, we provide a conclusion of this article in Section 5.

## 2. The Problem Formulation

### 2.1. The Basic Model of OFDM Power Allocation in CRNs

In this article, we consider the following application situation for IoT within a CRN. The service range of a base station covers one primary user and *K* secondary users. The primary user and the secondary users use adjacent frequency bands, and the secondary users use OFDM transmission technology. Moreover, we assume that the channel is slowly fading within one OFDM symbol period, and the base station has full channel state information. The total number of subcarriers is *N*, and the bandwidth of each subcarrier is $W_{c}$. Let $b_{k,n}$ be the transmission rate of user *k* on the subcarrier *n*. Thus, the maximum number of bits in a symbol transmitted on this subcarrier is set to [35,36,37]

(1) $$b_{k,n}=\left\lfloor \log_{2}\left\lfloor 1+\frac{p_{k,n}g_{k,n}^{2}}{\delta (N_{0}W_{c}+S_{k,n})}\right\rfloor \right\rfloor ,$$

where $\left\lfloor \cdot \right\rfloor$ denotes the floor function; $p_{k,n}$ represents the power of user *k* on subcarrier *n*; $g_{k,n}$ is the channel gain of user *k* on subcarrier *n*; and $N_{0}$ represents the noise spectral density power, which is the same for all users and subcarriers, and it is a constant. In addition, $S_{k,n}$ indicates the primary user’s interference to the secondary user. The variable δ indicates the BER of the transmission in the case of a physical layer using multiple quadrature amplitude modulation (MQAM) technology, and it can be expressed as

(2) $$\delta =-\frac{2}{3}\ln\left(5p_{e}\right),$$

where $p_{e}$ denotes the BER.

Generally, in a CRN, the optimization goal of power resource allocation is to maximize the total transmission rate of the system for subusers under the restriction of the authorized users’ interference threshold, total transmission power, and BER, so as to improve the spectrum utilization. Therefore, the problem can be modeled as

(3) $$\max\;W_{c}\sum_{n=1}^{N}\sum_{k=1}^{K}\lambda_{k,n}b_{k,n}=W_{c}\sum_{n=1}^{N}\sum_{k=1}^{K}\lambda_{k,n}\left\lfloor \log_{2}\left\lfloor 1+\frac{p_{k,n}g_{k,n}^{2}}{\delta (N_{0}W_{c}+S_{k,n})}\right\rfloor \right\rfloor ,$$

(4) $$s.t.\;\sum_{k=1}^{K}\lambda_{k,n}=1,\;\;\lambda_{k,n}=\left\{\begin{array}{c} 0,\;b_{k,n}=0\\ 1,\;b_{k,n}\neq 0 \end{array}\right.$$

(5) $$\sum_{n=1}^{N}\sum_{k=1}^{K}p_{k,n}⩽p_{\mathrm{total}}\;,$$

(6) $$\sum_{n=1}^{N}\sum_{k=1}^{K}\lambda_{k,n}p_{k,n}F_{n}⩽I_{\mathrm{th}}\;,$$

(7) $$p_{e}⩽p_{u}.$$

Here, Equation (4) shows that a subcarrier can only be occupied by one user. In addition, $\lambda_{k,n}$ is the subcarrier allocation state variable. It is equal to 1 only when the subcarrier *n* is occupied by user *k*; otherwise, it is equal to 0. Equation (5) shows that the sum of the power $p_{k,n}$ transmitted by all secondary users cannot exceed the total system upper power limit $p_{\mathrm{total}}$. In Equation (6), $F_{n}$ represents the interference factor of the secondary users to the primary user on subcarrier *n*, and $I_{\mathrm{th}}$ is the highest user-acceptable maximum interference limit. It means that all subusers’ interference to the primary user should not exceed its tolerable upper limit $I_{\mathrm{th}}$. Moreover, Equation (7) indicates that the BER must be less than the maximum BER requirement $p_{u}$ [38].

### 2.2. The Complex Model with User Rate Proportionality Constraints

Based on the practical scenario discussed above for the basic OFDM power allocation model in CRNs, instead of considering the primary interference constraints, we considered the fairness of channel resource allocation among secondary users. Then, the capacity of different secondary users needs to meet the certain rate proportional constraint, as follows [39].

(8) $$R_{1}:R_{2}:\cdots :R_{K}=\alpha_{1}:\alpha_{2}:\cdots :\alpha_{K},$$

(9) $$R_{k}=\sum_{n=1}^{N}\lambda_{k,n}b_{k,n},\;\;k=1,2,\cdots ,K$$

(10) $$R_{k}>0,\;\;k=1,2,\cdots ,K$$

In Equation (8), $\alpha_{k}\;\left(k=1,2,\cdots ,K\right)$ is a predefined constant, representing the rate proportional constraint that needs to be met by secondary users. Different users’ capacity is defined in Equation (9), and it can be further calculated with Equation (3). In addition, Equation (10) is a non-negative constraint on users’ capacity, which is implicitly indicated by Equation (8). In this model, we still strive for the maximum value of the defined function in Equation (3). The interference constraint, Equation (6), in the previous model is ignored, and we add constraints from Equations (8)–(10) to further limit the capacity of different secondary users.

## 3. The Proposed Solution Method Using the PA-Jaya Algorithm

The Jaya algorithm is a variant of swarm intelligence, and it achieves the optimal solution by constantly performing an iterative search of the same principle [33]. It has been verified that, in some cases, the Jaya algorithm is more flexible and more advantageous because of its parameterless feature. In an effort to avoid the computational limitations and further improve the performance, we propose an efficient solution method for the optimization problem in CRN-aided IoT, through the use of the PA-Jaya algorithm. To this end, in order to achieve faster convergence speed, PA-Jaya was developed by using a parallel structure and an asynchronous iteration strategy.

### 3.1. The General Idea of Jaya Algorithm

The Jaya algorithm was developed based on the thought that, during each iteration, the solution for a given problem should move toward the best solution and avoid the worst solution. Considering a general optimization problem, it is assumed that at any iteration *i*, the number of design variables is *N* and the population size is *M*. If $p_{m,n}^{\left(i\right)}≜p_{i,m,n}$ is the value of the *n*-th (n=1,2,⋯,2N) variable for the *m*-th (m=1,2,⋯,M) candidate during the *i*-th iteration, then this value is updated according to the following formula.

(11) $$p_{i,m,n}^{\prime }=p_{i,m,n}+r_{1}(p_{i,\mathrm{best},n}-|p_{i,m,n}\left|\right)-r_{2}(p_{i,\mathrm{worst},n}-|p_{i,m,n}\left|\right),$$

where $p_{i,\mathrm{best},n}$ is the value of the *n*-th parameter for the best solution and $p_{i,\mathrm{worst},n}$ is the value of the *n*-th parameter for the worst solution in the population. Moreover, $p_{i,m,n}^{\prime }$ is the new value of $p_{i,m,n}$, and $r_{1}$ and $r_{2}$ are two random numbers whose values are in the range of [0,1], respectively. The term “$r_{1}(p_{i,\mathrm{best},n}-|p_{i,m,n}\left|\right)$” indicates that the solution tries to move towards the best solution, and the term “$-r_{2}(p_{i,\mathrm{worst},n}-|p_{i,m,n}\left|\right)$” shows that the solution tries to avoid the worst solution. The two random numbers $r_{1}$ and $r_{2}$ provide the Jaya algorithm with the ability to perform a random search within the search space, theoretically ensuring that the algorithm can converge to the global optimal solution. In addition, the exploration capability of the Jaya algorithm is further improved by utilizing the absolute value of the candidate solution. $p_{i,m,n}^{\prime }$ is accepted if the function value it produces is better. Let $P_{i,m}={\left\{p_{i,m,n}\right\}}_{n=1}^{2N}$, $P_{i,m}^{\prime }={\left\{p_{i,m,n}^{\prime }\right\}}_{n=1}^{2N}$, and J(·) be the fitness evaluation function; then, it can be expressed as

(12) $$P_{i+1,m}=\left\{\begin{array}{cc} P_{i,m}^{\prime }, & \mathrm{if}\;J\left(P_{i,m}^{\prime }\right)>J\left(P_{i,m}\right)\\ P_{i,m}, & \mathrm{if}\;J\left(P_{i,m}^{\prime }\right)⩽J\left(P_{i,m}\right) \end{array}\right.$$

The Jaya algorithm updates the candidate solution by comparing the modified solution and its corresponding old solution. A given candidate solution is updated only when the fitness of its modified solution is better than that of the old one, in which case it is updated to the value of the modified solution. Otherwise, it keeps the value of the old one [33].

Afterward, the same procedure is followed for all the candidates in the population. Obviously, this means that only the best solutions are forwarded as input to the next iteration. In other words, the Jaya algorithm always tries to get closer to success (i.e., reaching the best solution) and tries to avoid failure (i.e., moving away from the worst solution). The algorithm struggles to become victorious by reaching the best solution: it is from this aspect that Jaya draws its name. The update procedure of the Jaya algorithm is shown in Algorithm 1. Compared with some traditional evolutionary algorithms, e.g., GA and PSO, there is no requirement for algorithm-specific parameters in Jaya, which is the biggest advantage of this algorithm [33,40,41,42,43].

**Algorithm 1** Jaya update procedure.
**Input:** population matrix *P*, population size *M*, the number of variables 2N, fitness vector *f*, the best and worst solution vectors: $p_{\mathrm{best}}$ and $p_{\mathrm{worst}}$
**Output:** updated population matrix *P*, updated fitness vector *f*

**for** m:=1toM **do**

 **for** n:=1to2N **do**
  Choose a random number $r_{m,n,1}$ from [0,1];
  Choose a random number $r_{m,n,2}$ from [0,1];
  $p_{m,n}^{\prime }=p_{m,n}+r_{m,n,1}(p_{\mathrm{best},n}-|p_{m,n}\left|\right)-r_{m,n,2}(p_{\mathrm{worst},n}-|p_{m,n}\left|\right)$;
 **end for**
 $f_{m}^{\prime }=J\left(P_{m}^{\prime }\right)$;
 **if** $f_{m}^{\prime }>f_{m}$ **then**
  **for** n:=1to2N **do**
   $p_{m,n}=p_{m,n}^{\prime }$;
  **end for**
  $f_{m}=f_{m}^{\prime }$;
 **end if**

**end for**



### 3.2. PA-Jaya for the Fundamental Issue in the Cognitive OFDM Radio Network

With the analysis mentioned in Section 2, the basic spectrum resource allocation problem discussed in this article can be modeled as a constrained optimization problem. Hence, the problem can be transformed into finding a user’s corresponding power allocation scheme for $p_{k,n}$ under the premise of satisfying the constraints in order to maximize the total transmission rate of all secondary users.

In the process of using evolutionary algorithms to solve problems, the coding representation of the individual population and the setting of the fitness function are crucial components. Therefore, here, we first specify these two parts.

According to the requirements mentioned above, the power allocation scheme can be expressed as a matrix of K×N dimensions. It can be expressed as

(13) $$P=\left[\begin{array}{ccccc} p_{1,1} & p_{1,2} & \cdots & p_{1,N-1} & p_{1,N}\\ p_{2,1} & p_{2,2} & \cdots & p_{2,N-1} & p_{2,N}\\ \vdots & \vdots & \vdots & \vdots & \vdots \\ p_{K,1} & p_{K,2} & \cdots & p_{K,N-1} & p_{K,N} \end{array}\right],$$

where the row of the matrix represents the user k(k=1,2,⋯,K), the column represents the subcarrier n(n=1,2,⋯,N), and each element of the matrix $p_{k,n}$ represents the power obtained by the user *k* on the subcarrier *n*. According to the constraint described by Equation (4), one subcarrier can be occupied by only one user; that is to say, there can be only one nonzero element per column of this matrix. Hence, we only considered those nonzero elements in the model.

**Figure 1:**
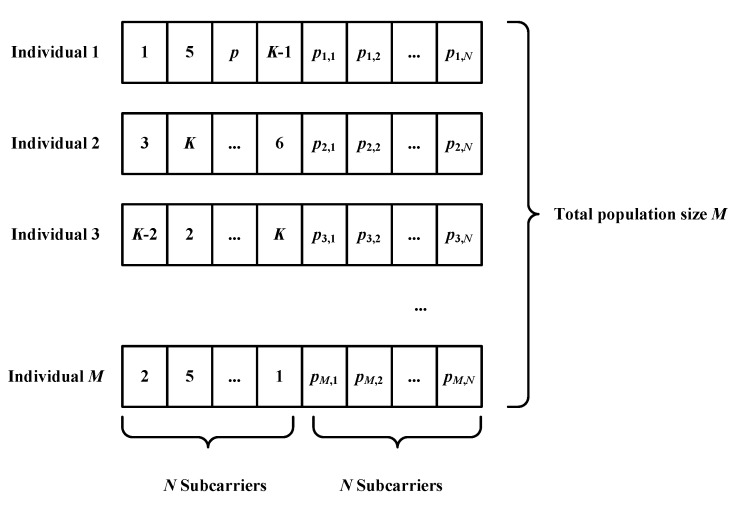
Structure of population and individuals designed for the power allocation problem.

Here, for convenience, we represent each solution individual in the population with a matrix of size 1×2N, in which the first *N* elements are in the range of [1,K], indicating how the *N* subcarriers are assigned to the *K* users. The next *N* elements represent the specific power values allocated to each subcarrier. According to Equations (5) and (6), these variables should be in the range of $\left[0,p_{\mathrm{total}}\right]$ and $\left[0,\frac{I_{\mathrm{th}}}{F_{n}}\right]$. Then, the structure of the population can be depicted as in Figure 1.

Since the basic spectrum resource allocation problem discussed in this article can be clearly expressed as a constrained maximization problem, we chose Equation (3) as the fitness evaluation function of the proposed PA-Jaya algorithm. Considering the specificity of the individual coding methods in this article, it is necessary to perform special processing when using the PA-Jaya algorithm for a population update. That is, the first *N* elements need to be rounded after each update, and they are bounded within [1,K]. The next *N* elements are only bounded within $\left[0,p_{\mathrm{total}}\right]$. The remaining constraints are judged after the whole population is generated, and the fitness value is set to 0 for the individual solutions that do not meet the requirements. All individual solutions need to be compared with the corresponding individual solutions before the population update, and those with larger fitness values enter the next iteration. Meanwhile, the best individuals and the worst individuals in the population are also updated.

The proposed PA-Jaya algorithm, as an improvement to the traditional Jaya algorithm, aims to speed up the convergence of the algorithm by updating the values asynchronously with a parallelized structure and by reducing infeasible solutions through an inner loop iteration strategy.

Motivated by the successful work of improving the convergence speed using asynchronous iteration in PSO [44,45], in the proposed PA-Jaya algorithm, the update of the best individual solutions is not performed only after the whole population update is completed, but is performed after each individual solution updates. Immediately after each individual solution update is completed, it is compared with all individual solutions of the previous generation, and the best individual is selected to update the subsequent individual solutions.

Another important improvement of the proposed Jaya algorithm is that it sets an inner loop to reduce the generation of infeasible solutions. Considering that the basic resource allocation problem in this article is a constrained optimization problem, all the candidate solutions obtained need to meet the requirements of Equations (5) and (6). In this case, if the traditional Jaya algorithm is directly applied, a large number of infeasible solutions will be generated in the population after each iteration, and these solutions will be discarded, resulting in unsatisfactory efficiency of the iterative solution. Therefore, an intuitive idea is to consider how to reduce these infeasible solutions during the update process of each iteration. The PA-Jaya algorithm addresses this problem through an inner loop in each iteration to repeat the update of a single candidate solution to satisfy the constraints as much as possible; thus, most of the solutions in the population after each iteration will be feasible solutions. Specifically, for the basic spectrum resource allocation problem discussed in this article, when we obtain the first candidate solution during the first iteration, we will immediately observe whether it meets the requirements of Equations (5) and (6). If these requirements are met, we continue to update the subsequent solutions in the population while also paying attention to the constraints, until all the candidate solutions are updated during this iteration. Otherwise, if the first candidate solution does not meet these constraints, the candidate solution will be regenerated until it meets all the requirements or the maximum number of inner loops is reached, and then the subsequent operations are consistent with the above. Thus, based on the above two tricks, the framework of the improved asynchronous Jaya algorithm can be described by Algorithm 2.

Furthermore, motivated by some successful work [46,47,48,49], we introduce the parallel structure into the proposed algorithm to make full use of computing resources and reduce the running time. Generally, parallel models of the common GA can be divided into four categories, including the master-slave model, coarse-grained model, fine-grained model, and hybrid model [47].

Here, we use the coarse-grained model, also called the land-based model, which is the most adaptable and widely used GA parallel model. The initial population is divided into several independent subpopulations, and the improved asynchronous Jaya iterative algorithm is used to perform evolution operations independently in each subpopulation. After a certain number of iterations, information exchange is conducted between different subpopulations, and the convergence speed is further accelerated by introducing the optimal individuals to the entire population. By adopting a parallelization structure, the independence between each subpopulation can be ensured, and the individual diversity of each subpopulation can be enriched. This not only ensures the exchange of excellent antibodies within the population, but also prevents the rapid spread of local optimal solutions and the occurrence of immature convergence. The update procedure of the improved PA-Jaya algorithm is shown in Algorithm 3.

**Algorithm 2** The improved asynchronous Jaya update procedure.
**Input:** population matrix *P*, population size *M*, the number of variables 2N, the number of inner loops *T*, fitness vector *f*, the best and worst solution vectors: $p_{\mathrm{best}}$ and $p_{\mathrm{worst}}$
**Output:** updated population matrix *P*, updated fitness vector *f*

**for** m:=1toM **do**

 **for** t:=1toT **do**
  **for** n:=1to2N **do**
   Choose a random number $r_{m,n,1}$ from [0,1];
   Choose a random number $r_{m,n,2}$ from [0,1];
   $p_{m,n}^{\prime }=p_{m,n}+r_{m,n,1}(p_{\mathrm{best},n}-|p_{m,n}\left|\right)-r_{m,n,2}(p_{\mathrm{worst},n}-|p_{m,n}\left|\right)$;
  **end for**
  **if** $P_{m}^{\prime }$ meets constraints (5) and (6) **then**
   break;
  **end if**
 **end for**
 $f_{m}^{\prime }=J\left(P_{m}^{\prime }\right)$;
 **if** $f_{m}^{\prime }>f_{m}$ **then**
  **for** n:=1to2N **do**
   $p_{m,n}=p_{m,n}^{\prime }$;
  **end for**
  $f_{m}=f_{m}^{\prime }$;
 **end if**

**end for**



**Algorithm 3** The improved PA-Jaya update procedure.
**Input:** population matrix *P*, the number of subpopulations *S*, the number of population iterations *D*, the number of iteration intervals of subpopulation information exchange *Q*
**Output:** updated population matrix *P*

**for** i:=1toD **do**

 **for** s:=1toS **do**
  Update $P_{s}$ with the improved asynchronous Jaya algorithm;
 **end for**
 **if** *i* mod *Q* = 0 **then**
  **for** s:=1toS **do**
   Replace the worst individual solution in $P_{s}$ with the global best individual solution;
  **end for**
 **end if**

**end for**



**Figure 2:**
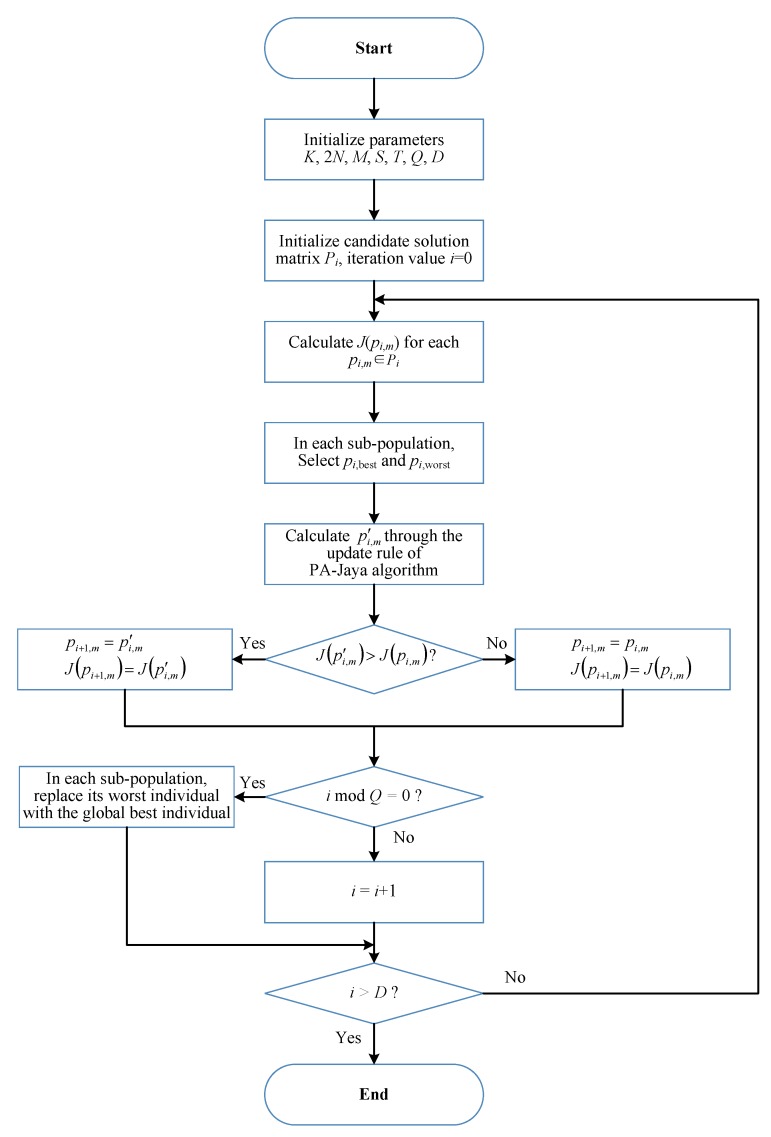
Flowchart of the PA-Jaya algorithm for the cognitive orthogonal frequency division multiplexing (OFDM) radio network power allocation model.

Figure 2 presents a schematic diagram for using the PA-Jaya algorithm to achieve optimization for the cognitive OFDM radio network power allocation model. The basic steps of the solution method are as follows.

**Step 1: Parameter setting.** It starts with the setting of initial parameters, including the number of secondary users (K), the number of variables (2N), population size (M), the number of population divisions (S), the number of inner loops (T), the number of iteration intervals of subpopulation information exchange (Q), and the algorithm termination criterion. For the current situation, the termination criterion is set as the maximum number of iterations (D), which means that the algorithm is terminated when it iterates more than this value.

**Step 2: PA-Jaya initialization.** Initial values of individual solutions can be randomly generated under the constraints in Equations (5) and (6). After finishing the same operation for all the variables in the whole population, the solutions matrix is well initialized.

**Step 3: Fitness evaluation.** Once all the candidate solutions are initialized, every individual solution is evaluated with the fitness function. Considering the practical problem in this article, the fitness function’s purpose is to get the maximum object value according to Equation (3).

**Step 4: Solution update.** In each parallel subpopulation, by comparing the fitness function value of each candidate solution, we can easily select the best and the worst solutions. Hence, we are able to modify the old solution with the proposed asynchronous Jaya iteration strategy. Let $P_{i,m}={\left\{p_{i,m,n}\right\}}_{n=1}^{2N}$, $P_{i,m}^{\prime }={\left\{p_{i,m,n}^{\prime }\right\}}_{n=1}^{2N}$, and let J(·) be the mathematical operation in Equation (3), respectively. Then, the update of the candidate solution is checked by assessing

(14) $$J\left(P_{i,m}^{\prime }\right)>J\left(P_{i,m}\right).$$

If Equation (14) is met, $p_{i,m}^{\prime }$ will be applied to the basic power allocation model; that is to say, the candidate solution will be updated through $p_{i+1,m}=p_{i,m}^{\prime }$. Otherwise, it retains the original value. Generally, the traditional Jaya updates its best and worst individuals after the entire population has been updated. Compared with this, PA-Jaya updates the best value in every single individual calculation, and it can be seen as an asynchronous update strategy. In addition, a maximum number of loops in the inner layer is set to reduce infeasible solutions with the purpose of further accelerating the speed of convergence in PA-Jaya. Thus, the PA-Jaya algorithm guides the candidate solution update in each independent subpopulation, while all these subpopulations exchange information periodically. The whole process can be described by Algorithm 3.

**Step 5: Convergence criterion.** The stopping condition is checked. Once the searching process reaches the maximum number of iterations, the loop is terminated and the optimum solution is obtained.

### 3.3. PA-Jaya for the User Fairness Issue in Cognitive OFDM Radio Network

This problem becomes more complicated when the secondary user rate proportional constraint is considered. Obviously, in this problem, the subchannel and power should be jointly allocated to achieve the optimal capacity under the user rate proportional constraints. However, this joint optimization results in high computational costs. In order to address this complex model, the traditional methods are usually based on three steps. The first step is to allocate channels for each secondary user based on the interference factors of each channel. The second step roughly allocates the total transmit power to each user based on the number of channels obtained by each secondary user. The third step is implemented on the basis of the previous two allocations while using the traditional water-filling algorithm to choose the best allocation method for every secondary user.

In order to better handle this problem, the following fairness indicator is introduced [50].

(15) $$I={\left(\sum_{i=1}^{K}\frac{R_{k}}{\alpha_{k}}\right)}^{2}{\left(K\sum_{i=1}^{K}{\left(\frac{R_{k}}{\alpha_{k}}\right)}^{2}\right)}^{-1}.$$

The maximum value of Equation (15) is 1, and when it reaches this value, the fairness of the allocation model is optimal.

The signal-to-noise ratio (SNR) in this model can be written as

(16) $$\mathrm{SNR}=\frac{g_{m,n}^{2}}{\delta (N_{0}W_{c}+S_{m,n})}.$$

Theoretically, allocating more power to the channels with larger SNRs can maximize the total channel capacity demanded in this model, but doing so may not satisfy the user fairness constraints in Equation (10).

Then, considering the user fairness issue, an optimization approach, called the ROOT-FINDING algorithm, was proposed on the basis of proportional rate constraints [39]. For the case of high channel gain, the ROOT-FINDING algorithm can solve the user rate proportional constraint problem well and achieve spectrum efficiency improvement. However, in order to satisfy the proportional constraint, many subcarriers are often not correctly allocated to the optimal user, resulting in a low overall utilization efficiency of the subcarrier. In Ref. [51], an improved algorithm, called LINEAR, was proposed in an effort to effectively address this problem. This algorithm achieves an increase in spectral efficiency by relaxing the fairness constraint while improving the computational time. Since the user fairness and spectral efficiency may not be well weighed, the algorithm still has room for improvement.

In response to these limitations, through the use of the two-stage allocation scheme described in Ref. [51], here we present a novel method on the basis of the PA-Jaya algorithm. In the first stage, we fully consider the state of each channel. Assuming that transmit power is evenly distributed across all subchannels, then we can reasonably suppose that the proportion of subcarriers assigned to different users is approximately the same as their capacity rates after power allocation. Then, we use the proposed PA-Jaya algorithm to allocate each channel to the user with the best SNR as much as possible while satisfying the total power constraints and user fairness constraints. This procedure can be described as follows.

First, we assign each secondary user the unallocated subcarrier that has the maximum SNR for that user. Then, each user is assigned a number of subcarriers according to his proportion constant, and the priority of the secondary users is determined by the ratio of his obtained capacity to the proportion constant, which is $\frac{R_{k}}{\alpha_{k}}\;\left(k=1,2,\cdots ,K\right)$. Finally, all unoccupied subcarriers are allocated to users who can obtain the maximum SNR; in this case, each secondary user can get one subcarrier at most.

After the subcarrier allocation procedure, we use the proposed PA-Jaya algorithm to further distribute power. Motivated by the work in Ref. [50], in order to better apply our proposed PA-Jaya algorithm, we designed a special fitness function for this issue, which is

(17) $$F=\left\{\begin{array}{cc} \;W_{c}\sum_{n=1}^{N}\sum_{k=1}^{K}\lambda_{k,n}b_{k,n}\; & \mathrm{if}\;I⩾\epsilon \\ \;I-\epsilon \; & \mathrm{otherwise} \end{array}\right.$$

Here, ϵ is a predefined constant representing the minimum requirement for the fairness indicator. In this function, it is obvious that for those solutions which do not meet the user fairness constraint, the algorithm selects them through the fitness function so that they can gradually move in the direction of better satisfying the user fairness requirement. For those solutions satisfying the requirement, the algorithm helps them acquire more channel capacity, eventually converging to the optimal solution. The introduction of ϵ makes it possible to maximize the channel capacity while satisfying the minimum user fairness requirement. Moreover, the specific algorithm structure is similar to the previous one; it is not described in detail here.

### 3.4. Computational Complexity Analysis for PA-Jaya

As described above, the population size is set as *M*, the number of variables in each candidate solution is 2N, and the maximum number of inner iterations is *K*.

According to the flow shown in Algorithm 2, it is obvious that our proposed PA-Jaya algorithm needs to use a triple loop for each iteration update. The number of the outermost loop is set as the population size *M*, which indicates that each individual solution in the population is traversed to ensure that all solutions in the population are updated. The number of the middle loop is set as a predefined constant *K*. The function of this loop is to minimize the generation of infeasible solutions in the updated population, so as to accelerate the overall convergence speed of the algorithm. The number of the innermost loop is set as the number of variables 2N in each candidate solution. This loop is essential because our PA-Jaya algorithm needs to generate two random numbers, $r_{1}$ and $r_{2}$, for each of these variables when updating each candidate solution.

Then, in the worst case during each iteration, the time complexity required for our proposed PA-Jaya algorithm is O(M×N×K), which seems to be *K* times the time required by the traditional Jaya algorithm. However, considering that *K* is a relatively small constant, such an increase in time is acceptable. In addition, PA-Jaya requires fewer iterations to converge, which also makes up for the increase in time required for each iteration to some extent. Moreover, by using a parallelized structure, our algorithm makes full use of existing computing resources while greatly reducing the runtime of the algorithm.

## 4. Simulation Results and Analyses

### 4.1. Simulation Setup

The simulation was conducted in the environment of MATLAB/Win 7. Assuming that the CRN-aided IoT system exists with a multipath frequency selective fading channel, and that the channel gain of each subcarrier is subject to Rayleigh fading with an average channel gain of 1, then the parameters of the simulation are set as shown in Table 1.

In order to verify the performance of our proposed solution method using the PA-Jaya algorithm, the simulation model was run 10 times under the same parameter settings, and the average value was obtained. Moreover, the computational performance was also compared between our solution method and other popular algorithms, including SA [20], GA [21], PSO [22,23], DE [24], ICO [25], and traditional Jaya [33]. The detailed parameter settings for each algorithm can be found in Table 2. It is noted that for our proposed PA-Jaya algorithm, the initial population is randomly generated, and each subpopulation is updated by asynchronous Jaya iteration, as shown in Algorithm 2. Then, information exchange between different subpopulations takes place at regular intervals, and the overall process can be seen in Algorithm 3.

Moreover, for the user fairness issue discussed in this article, the user fairness proportion factor is randomly generated. Then, we compare our proposed PA-Jaya algorithm with the algorithms ROOT-FINDING [39] and LINEAR [51]. Our solution to this issue can be divided into two parts. The first part is the allocation of subchannels, which is the same as that in Ref. [50]. In the second part, we use the PA-Jaya algorithm to allocate the appropriate power to the channels of different users while meeting the required constraints. As the fitness function is set as Equation (17), and the minimum user rate proportionality is set to 0.99, the process of conducting PA-Jaya and setting the parameters are consistent with the former issue.

### 4.2. Results and Discussion

**Figure 3:**
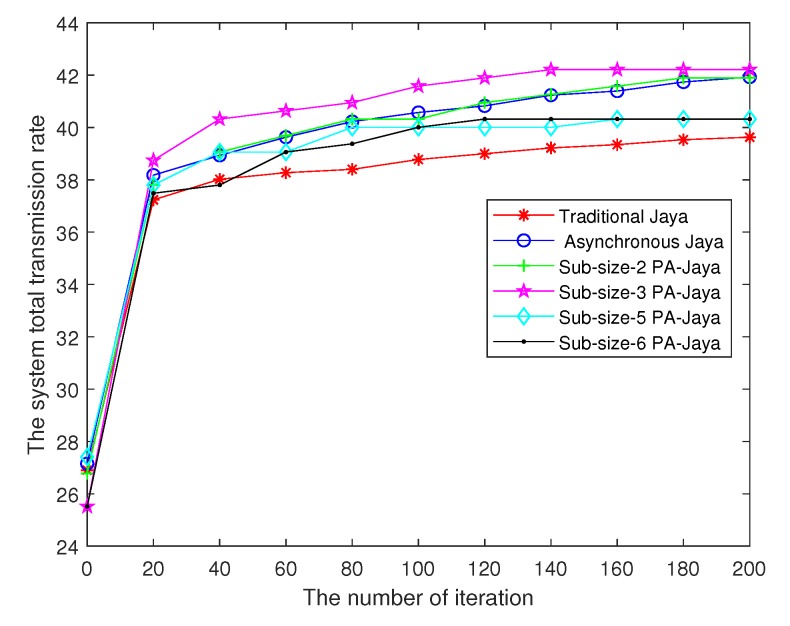
Performance comparison of different Jaya algorithms.

We first compare the results of our proposed PA-Jaya algorithm in different subpopulation size cases with the asynchronous Jaya and traditional Jaya algorithms. When the total transmit power ($p_{\mathrm{total}}$ = 1 W) and the BER (δ = 5 dB) are limited (which means that the model constraints are satisfied), and the maximum number of iterations *D* is reached, the results of different algorithms are as shown in Figure 3.

In Figure 3, the PA-Jaya algorithm can achieve better results compared with the traditional Jaya algorithm and the asynchronous Jaya algorithm without a parallel structure. Meanwhile, compared with the traditional Jaya algorithm, several other improved Jaya algorithms can get better results, which could be due to the way that asynchronous iteration improves the convergence speed. In addition, comparing the asynchronous Jaya algorithm and the PA-Jaya algorithm with different subpopulation sizes, it can be seen that when the number of subpopulations is 2, the result of PA-Jaya is similar to that of the asynchronous Jaya algorithm. When the number of subpopulations is 3, the result of PA-Jaya is better. However, as the number continues to grow, the result gets worse. On the one hand, this phenomenon shows that the use of a parallel structure can increase the diversity of the whole population to prevent premature local convergence, thereby further improving the performance of the Jaya algorithm. On the other hand, it also shows that when the subpopulation size is too small, the performance of the algorithm is limited. Taking into account the above situation, we set the number of subpopulations to 3 for the simulations hereafter reported in this article.

**Figure 4:**
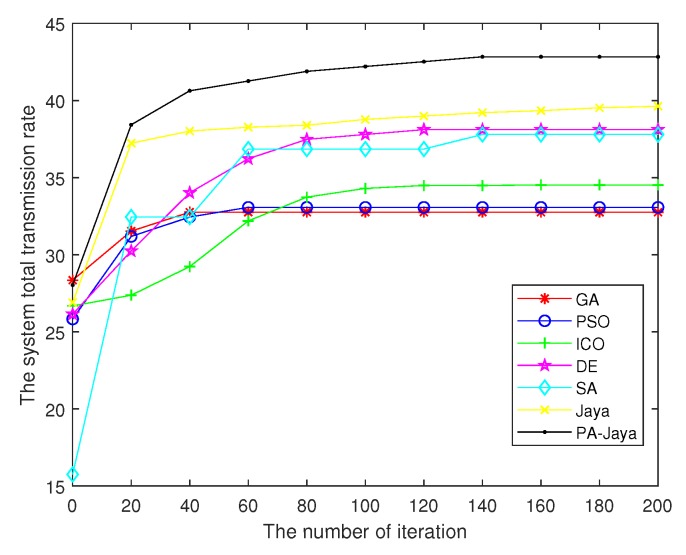
Relationship between the number of iterations and the total system transmission rate.

**Figure 5:**
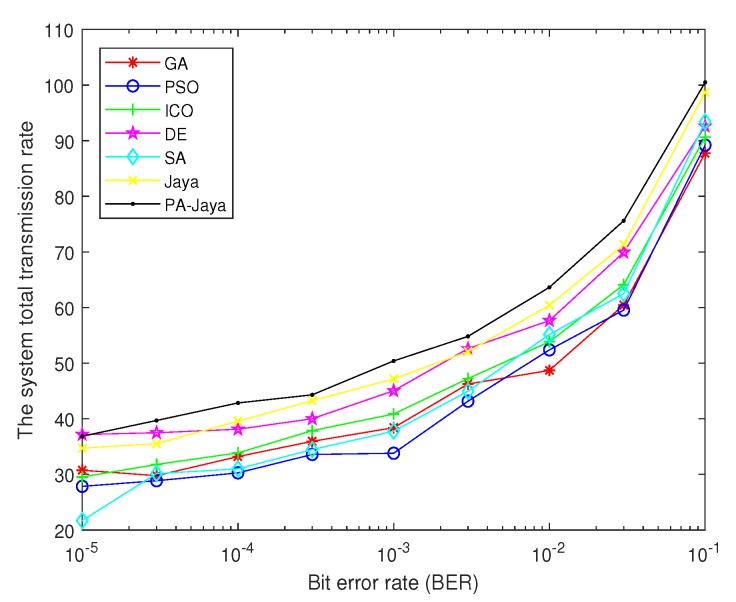
Relationship between the bit error rate (BER) and the total system transmission rate.

**Figure 6:**
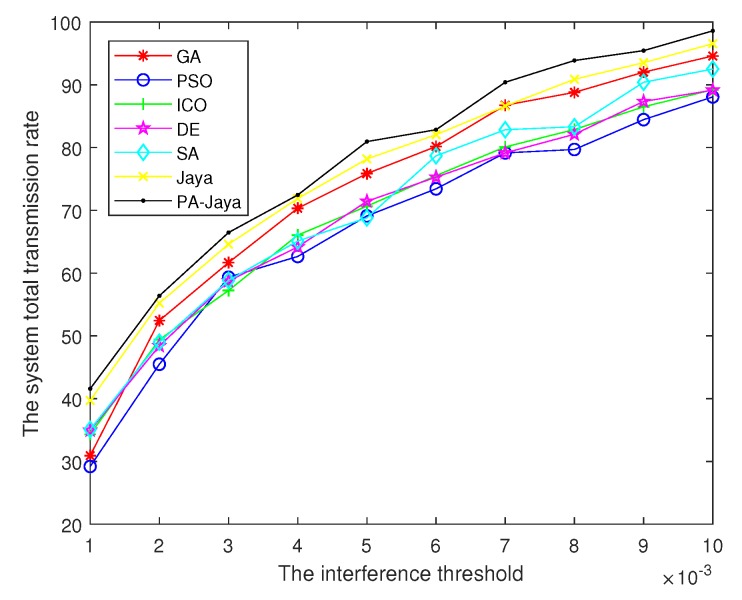
Relationship between the interference threshold and the total system transmission rate.

Meanwhile, Figure 4 shows the total transmission rate of the secondary users obtained by those algorithms under the same conditions. Then, when the number of secondary users is 8 and the maximum number of iterations *D* is reached, the total transmission rate of the system under different BERs is as shown in Figure 5. Furthermore, the optimal solutions of the total system transmission rate are shown in Table 3, and they are recorded in every 20 generations. While setting the BER of the transmission (δ) and the maximum number of iterations (*D*) to 5 dB and 200, respectively, the total transmission power of the secondary users changes as shown in Figure 6 with the interference tolerance of different primary users.

From Figure 4, it can be seen that, in each iteration, the solution performance of the total system transmission power obtained by our proposed PA-Jaya algorithm is obviously better than that of SA, GA, PSO, DE, ICO, and traditional Jaya algorithms while also achieving faster convergence speed. On the one hand, this is because of the iterative method and parameterless feature of the Jaya algorithm, which make it superior to other algorithms and allow it to converge faster. Then, the asynchronous iteration method we use further accelerates the process. In addition, it can be seen from this figure that several other algorithms fall into local convergence prematurely, and our PA-Jaya algorithm can avoid it well by adopting a multiple-subpopulation structure. This parallel structure helps our PA-Jaya algorithm keep searching for the optimal solution during the iteration.

Moreover, as shown in Figure 5, with the lower BER required by the system, the constraint requirements decrease, so the total system transmission rate increases. It also indicates that the system can effectively adapt to different BER constraints in the power allocation model. For the same reason, the solution performance of our PA-Jaya algorithm is also better than those of SA, GA, PSO, DE, ICO, and the traditional Jaya algorithms.

Figure 6 shows that with the increase of the tolerable interference threshold, the transmission power allowed to be used by the secondary users increases, and, therefore, the total transmission power of the system increases. Meanwhile, with the increase of the main user’s tolerable interference threshold, our algorithm achieves better performance for the same reason mentioned above.

**Figure 7:**
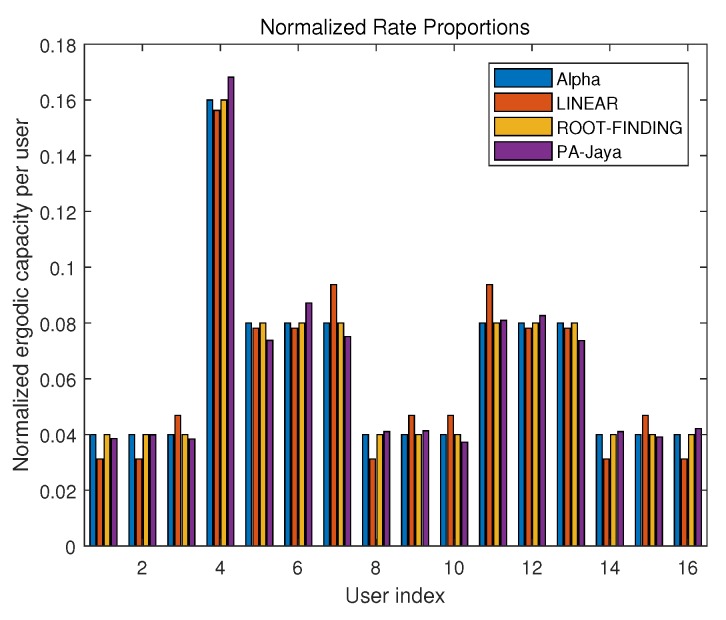
Comparison of different algorithm effects under normalized rate proportional constraint.

**Figure 8:**
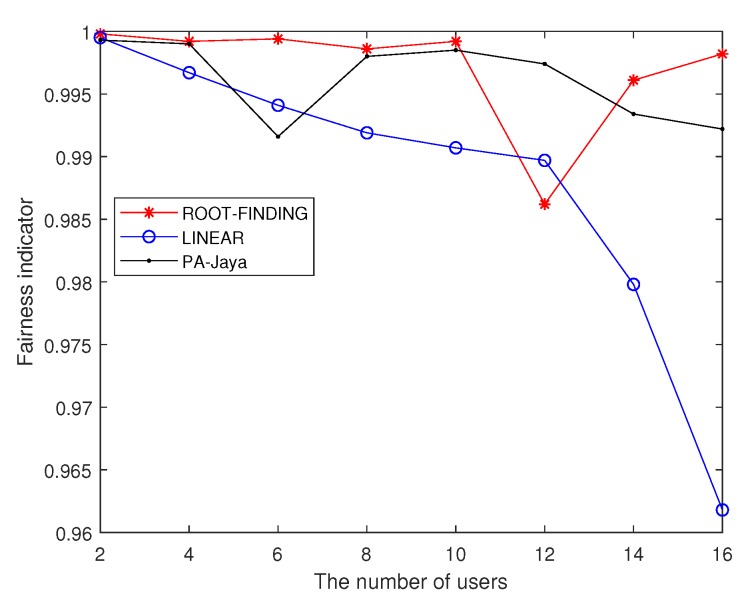
Comparison of different algorithms for the fairness indicator with different user numbers.

**Figure 9:**
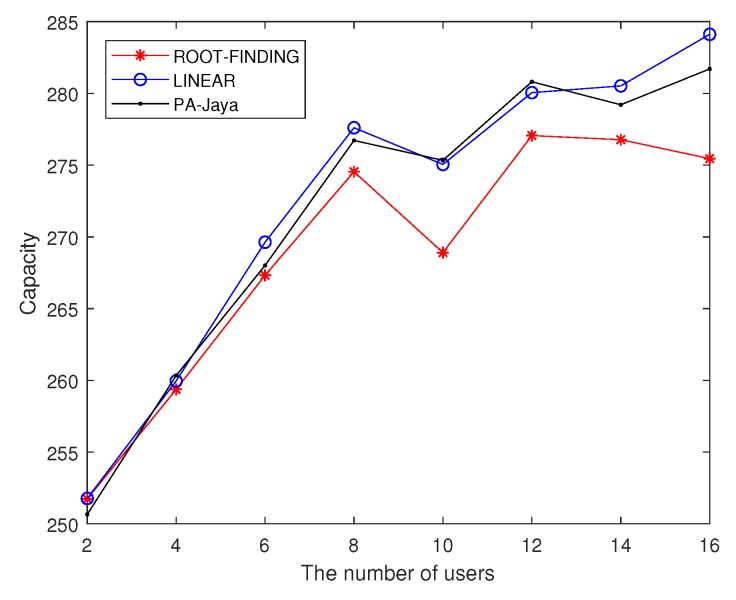
Comparison of different algorithms for capacity with different user numbers.

Subsequently, with the same settings for the simulation parameters, we used the proposed PA-Jaya algorithm to solve the multi-user OFDM CRN problem with the user proportional rate constraints mentioned in Section 2.2. Here, the interference generated by the secondary users to the primary users is ignored, and the proportional rate between different users is randomly generated. We set the number of secondary users as 16, with the purpose of better comparing the result of our PA-Jaya algorithm and others in the case of a large number of secondary users, which is slightly different from the former simulation setup. In Figure 7, the comparison is clearly shown, where “Alpha” indicates the user proportionality factor required in this model. Figure 8 and Figure 9 show the comparison of these different algorithms in the user fairness indicator (I) and channel capacity with different user numbers, respectively.

From the above simulation results, it is obvious that as the number of users increases, it becomes more difficult for the ROOT-FINDING algorithm to make full use of channel resources, so the channel capacity cannot achieve its optimal value. Meanwhile, the LINEAR algorithm has difficulty meeting the user fairness requirement. Our proposed PA-Jaya algorithm makes a compromise between these two aspects by adopting the fitness function and minimum user fairness constraint set. PA-Jaya achieves a similar result to LINEAR in terms of capacity. It is also shown that with the PA-Jaya algorithm, we can better weigh the capacity and fairness indicator, thus obtaining better results than the other algorithms.

## 5. Conclusions

In this article, with the aim of applying the CRN-aided IoT to the CPSS, a solution method for multi-user cognitive OFDM radio network power allocation using our PA-Jaya algorithm is proposed. In consideration of the special advantages of the swarm intelligence-based PA-Jaya algorithm for tackling the optimization problem with faster convergence and higher efficiency, the solution method using PA-Jaya may be a competitive choice for addressing the power allocation issue in CRN-aided IoT with high-quality requirements for complex parameter optimization. Simulation results show that the proposed algorithm is an efficient scheme for CRNs and can maximize the total transmission rate of this system model while satisfying the requirements of tolerable interference, total power limitation, and BER of the primary user. For user fairness issues, our proposed PA-Jaya algorithm can maximize the required channel capacity while satisfying certain user fairness constraints. By employing our PA-Jaya algorithm, the CRN technology is further enhanced to sense environmental changes and adaptively adjust its transmission parameters so that it works well in WSN systems, which has important practical value for production scenarios.

## Figures and Tables

**Table 1:** Model parameters.

Description	Parameter	Value
BER	$p_{e}$	$10^{-5}$−$10^{-1}$
Transmit BER	δ	5 dB
Noise spectral density power	$N_{0}$	$10^{-7}$ W/Hz
Interference factor	$S_{k,n}$	$10^{-6}$ W
Subcarrier bandwidth	$W_{c}$	0.315
Total system upper power limit	$p_{\mathrm{total}}$	1−30 W
User-acceptable maximum interference limit	$\frac{I_{\mathrm{th}}}{F_{n}}$	$10^{-3}$−$10^{-2}$ W
The number of secondary users	*K*	8
Population size	*M*	30
Total number of subcarriers	*N*	64
The number of subpopulations	*S*	3
The iteration interval of subpopulation communicate	*Q*	10
The number of inner loop	*T*	10
Total number of iterations	*D*	200

**Table 2:** Controlled parameters of PA-Jaya and other comparative algorithms.

Parameter	SA	GA	PSO	DE	ICO	Jaya	PA-Jaya
Initial temperature	100	–	–	–	–	–	–
Reannealing interval	100	–	–	–	–	–	–
Population size	–	30	30	30	30	30	30
Scaling factor	–	–	–	0.3	–	–	–
Crossover factor	–	0.3	–	0.1	0.3	–	–
Mutation factor	–	0.1	–	–	0.1	–	–
Initial inertia weight	–	–	0.9	–	–	–	–
Convergence inertia weight	–	–	0.4	–	–	–	–
Local acceleration constant	–	–	2	–	–	–	–
Global acceleration constant	–	–	2	–	–	–	–
Cloning proportion	–	–	–	–	0.2	–	–

**Table 3:** Optimal solutions of the total system transmission rate during iterations.

Generation	GA	PSO	ICO	SA	DE	Jaya	PA-Jaya
0	28.35	25.83	26.68	15.75	26.15	26.90	27.04
20	31.53	31.19	27.37	32.45	30.24	37.23	38.43
40	32.76	32.45	29.23	32.45	34.02	38.02	40.01
60	32.76	33.08	32.19	36.86	36.23	38.27	41.27
80	32.76	33.08	33.74	36.86	37.49	38.40	41.90
100	32.76	33.08	34.30	36.86	37.80	38.78	42.21
120	32.76	33.08	34.50	36.86	38.12	39.00	42.53
140	32.76	33.08	34.50	37.80	38.12	39.22	42.84
160	32.76	33.08	34.52	37.80	38.12	39.34	42.84
180	32.76	33.08	34.52	37.80	38.12	39.53	42.84
200	32.76	33.08	34.52	37.80	38.12	39.63	42.84
